# Subtotal Parathyroidectomy and Relocation of the Parathyroid Remnant for Renal Hyperparathyroidism: modification of a traditional operation

**DOI:** 10.1186/s40463-017-0238-7

**Published:** 2017-10-23

**Authors:** Tsu-Hui ( Hubert) Low, John Yoo

**Affiliations:** 1 Head and Neck Department, Chris O’Brien Lifehouse, Missenden Road, Camperdown, NSW Australia; 2 Department of Otolaryngology-Head and Neck Surgery, London Health Sciences Centre, Schulich School of Medicine& Dentistry, Western University, 800 Commissioners Road East, Suite B3-433A, London, ON N6A 5W9 Canada

**Keywords:** Parathyroidectomy, Secondary hyperparathyroidism, Renal failure, Recurrence, Hypercalcaemia

## Abstract

**Background:**

We describe a modification of the conventional subtotal parathyroidectomy operation where the parathyroid gland(s) remnant is repositioned with intact vascular supply to a plane superficial to the infrahyoid strap muscles and immediately under the skin incision. This technique called Subtotal Parathyroidectomy and Remnant Relocation (SPARE) retains all the metabolic advantages of the conventional operation with the added advantage of easier identification of a recurrent hyperplastic remnant if re-exploration becomes necessary.

**Methods:**

In the SPARE technique, four parathyroid glands were identified and the quality of each gland and the length of each vascular pedicle to the parathyroid glands were assessed. The optimal parathyroid gland was relocated to a plane superficial to the strap muscles. The remainder of the glands were removed.

**Results:**

In total, 30 patients with hyperparathyroidism secondary to renal failure (HSRF) underwent parathyroidectomy with the SPARE technique. The mean age was 53.1±12.5 years and median follow-up was 17.1 months (range 1-78.9 months). There were no recurrent laryngeal nerve (RLN) injuries or hematomas. The pre- and post-operative value for corrected calcium and PTH were 158.4±109.4 pmol/L and 11.4±12.1 pmol/L, respectively (*p* < 0.05). Three recurrences were noted (10.0%), with a mean time to recurrence of 15.3±6.6 months. One patient had excision of the remnant parathyroid glands performed under local anaesthetic (29 min); one had re-exploration performed under general anaesthetic (81 min), and one was managed medically.

**Conclusion:**

We described a novel parathyroidectomy technique for patients with HSRF, which provides the advantages of conventional subtotal parathyroidectomy while mitigating the challenges of thyroid bed re-exploration when recurrences arise.

## Background

Hyperparathyroidism from hyperplasia of the parathyroid glands is common amongst patients with renal failure on long-term dialysis [1, 2]. Uncontrolled hyperparathyroidism negatively impacts the health and quality of life of the patients [3]. Up to 20% of the patients with hyperparathyroidism secondary to renal failure (HSRF) are referred for surgery due to failure of medical treatment [3]. Common indications for surgery include elevated levels of parathyroid hormone (PTH), hypercalcaemia (>2.6 mmol/L), hyperphosphataemia (>1.95 mmol/L), bone disease (osteitis fibrosa cystica on X-ray), severe symptoms (pruritus, malaise, bone pain), progressive ectopic calcification, and calciphylaxis [4]. Parathyroidectomy is the standard treatment for HSRF and is shown to improve symptoms, bone and mineral metabolism, cardiovascular risk factors, and overall quality of life [5, 6].

Three major parathyroidectomy approaches have been widely utilized for patients with HSRF. These include subtotal parathyroidectomy (STP), total parathyroidectomy with autotransplantation (TPAT) and total parathyroidectomy without autotransplantation (TP) [1]. In STP all hyperplastic parathyroid glands are removed except for a small remnant of vascularized parathyroid tissue (50 mg), which is left inside the thyroid bed [7, 8]. The blood supply and neurovascular relationship of the parathyroid glands has been well described. There is considerable variability but most commonly arise from branches of the inferior thyroid artery [9, 10]. The main advantage of STP is the maintenance of the native blood supply to the remnant parathyroid gland, hence preserving the ability of the remnant gland to continue secreting PTH hormone. This reduces the severity and duration of symptomatic hypocalcaemia that may result in the post-operative period [8, 11]. An additional advantage of this technique may be a lower incidence of recurrences as compared to TPAT [12]. The major disadvantage with STP is the difficulty in re-exploration of the thyroid bed for recurrent disease, with the potential of injury to the RLN [8]. Unfortunately, with continued dialysis, recurrence of HSRF is a frequent occurrence regardless of initial surgical technique, and may be caused by proliferation of the remnant or autotransplanted parathyroid tissue or a supernumerary gland [13].

TPAT may mitigate the need for thyroid bed re-exploration when recurrent disease occurs because parathyroid glands may be autotransplanted into different sites such as the sternocleidomastoid muscle or brachioradialis muscle of the forearm [14, 15]. However a disadvantage may be a higher rate of recurrence and the difficulty in managing these cases. On a recent meta-analysis the rate of recurrent disease was found to be higher for TPAT as compared to STP [12]. In addition, there is often a delay in return of the parathyroid function since the autotransplanted parathyroid tissue requires recruitment of new blood supply from the host bed to regain function [8].

In recent years, some surgeons have adopted the TP for patients with HSRF [7, 16]. Several studies have noted the return of PTH levels in some patients over time despite complete removal of the parathyroid gland [16–18]. Series utilizing TP have noted reduced rates of recurrent hyperparathyroidism [19] as compared to both STP and TPAT. However the likelihood of permanent hypoparathyroidism is significantly higher with TP [18, 19] and for this reason is generally a technique that is offered for selected patients.

STP may be the most appropriate procedure for patients who are candidates for renal transplantation or for patients with persistent hyperparathyroidism following renal transplantation (tertiary hyperparathyroidism). Recent series comparing TP, TPAT, and STP in patients who went on to renal transplantation found that patients who underwent STP demonstrated better kidney function during the post-operative period [11, 20].

The objective of this study was to describe a technical modification of the conventional STP operation. **S**ubtotal **p**arathyroidectomy **a**nd **re**location of the parathyroid remnant (SPARE) preserves a vascularized parathyroid remnant, identical to the STP but this remnant is repositioned to a more accessible location superficial to the plane of the infrahyoid strap muscles. The SPARE modification retains all the metabolic advantages of STP while enabling easier revision surgery and reducing the risk associated with thyroid bed re-exploration in the event that the remnant becomes the source of recurrence.

## Methods

### Surgical technique

SPARE technique began with a standard collar incision, retraction of the infrahyoid strap muscles, and exploration to identify all the hyperplastic parathyroid glands. The degree of hyperplasia was assessed by size, nodularity and firmness.

**Fig. 1:**
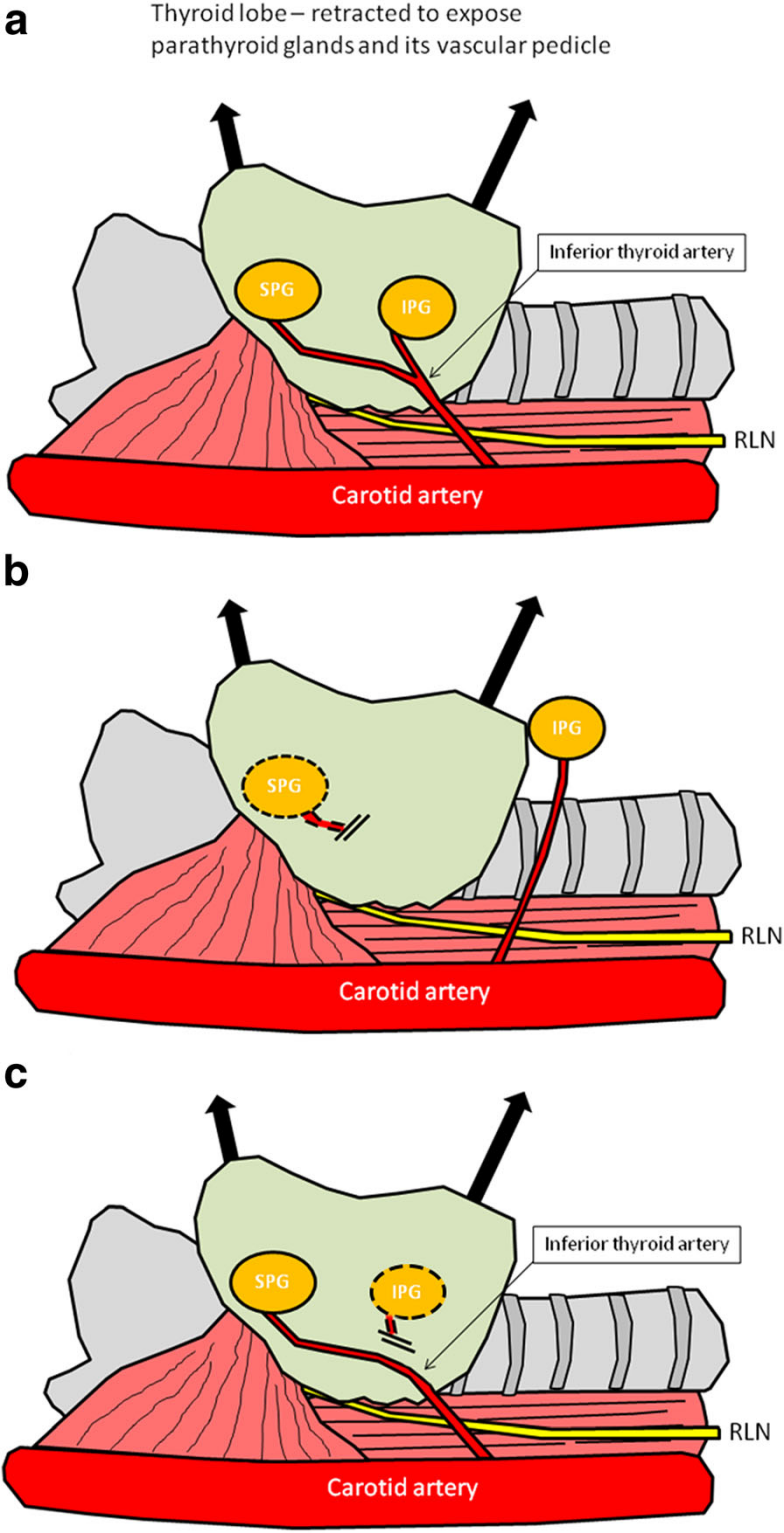
Schematic diagram of the relocating procedure of either the IPG or SPG. **a Legend:** represents the typical location of the superior (SPG) and inferior (IPG) parathyroid glands supplied by inferior thyroid artery (ITA) and their relationship with the recurrent laryngeal nerve (RLN). **b Legend:** represents the relocated position of the new IPG. The SPG was removed after ligating the vascular input from the ITA. The IPG is relocated to a new plane by mobilizing the inferior thyroid pedicle to allow positioning without excessive tension on the vascular pedicle. **c Legend:** represents the relocated position of the new SPG. The IPG was removed after ligating the vascular input from the ITA. The SPG is relocated to a new plane by mobilizing the superior thyroid pedicle to allow positioning the SPG usually directly through and superficial to the strap muscles

The parathyroid glands and vascular pedicles of each gland were assessed to determine which gland may be appropriate for relocation to a plane superficial to infrahyoid strap muscles. Once the gland with the most ideal pedicle length was identified and dissected, the other hyperplastic glands were removed and confirmed on frozen section (Fig. 1a, b, and c).

**Fig. 2:**
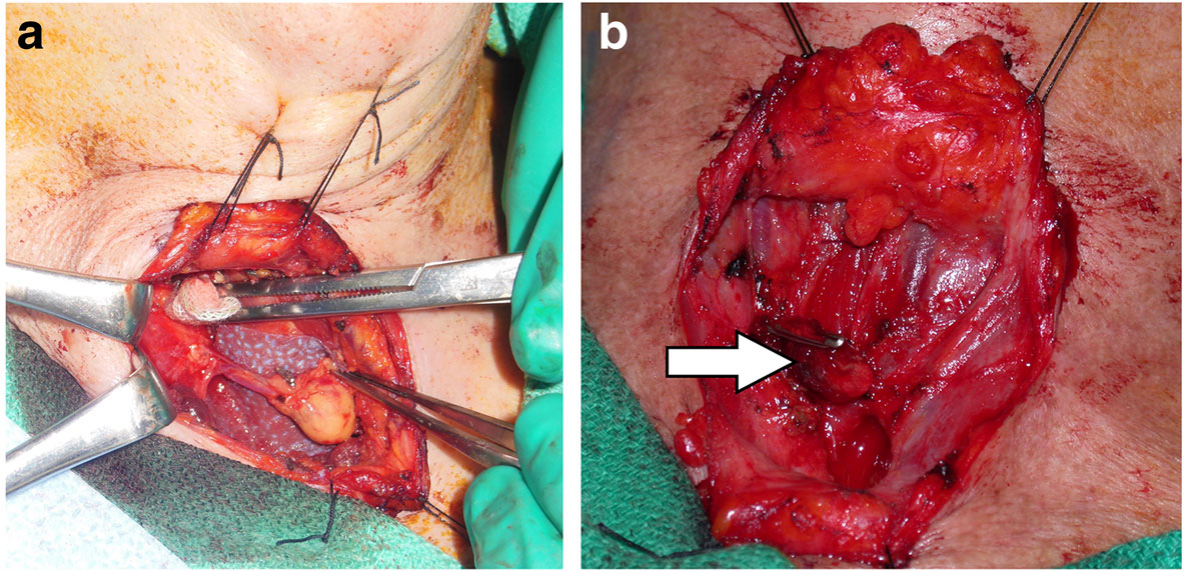
Intra-operative photo of the mobilized right inferior parathyroid gland on its vascular pedicle. **a Legend:** Lower parathyroid gland pedicled on the ITA. **b Legend:** Lower gland pedicled between strap muscles

When an inferior gland was selected for preservation, it was subsequently repositioned directly at the midline, between the divided midline raphe, and superficial to the strap muscles. The strap muscles were re-approximated with sutures while the parathyroid was held in position between the strap muscles. This prevented the preserved parathyroid gland from retracting inward (Fig. 2a and b).

**Fig. 3:**
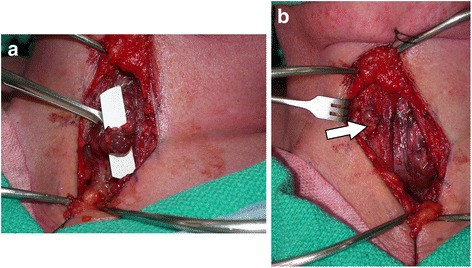
Intra-operative photo of the final position of the parathyroid gland. **a Legend:** Upper parathyroid gland pedicled on the ITA. **b Legend:** Upper gland directly through strap muscle

On occasion, the vascular pedicles for any of the parathyroid glands were too short for ideal midline relocation. In particular this usually occurred with preservation of the superior gland. In order to reduce tension of the vascular pedicle, a small perforation through the strap muscle directly above the preserved parathyroid gland enabled relocating the gland in the appropriate superficial plane (Fig. 3a and b).

The viability of the relocated parathyroid gland was then reassessed. A portion of the gland was then removed so that approximately 50 mg of parathyroid remnant remained. In doing so, bleeding from the remnant confirmed vascularity. The remnant was then marked with 2-3 large metal clips for easier future identification. Skin closure was carried out in the standard fashion and drains were not routinely inserted.

Depending on the quality of the parathyroid glands, more than one remnant may be saved. If necessary, a partial thyroidectomy may improve positioning the pedicled remnant. Routine removal of thymus was not performed if four parathyroid glands were found on initial exploration.

### Peri-operative and post operative management

The calcium management was carried out in collaboration with the renal team. When possible, patients were preloaded with calcium supplementation and dialysed before the surgery. The corrected calcium and PTH levels were checked while patients were in the recovery room and the first postoperative day. The patients were routinely started on oral calcium replacement. The calcium levels were monitored every 6 h for the first 24 h and calcium corrected as required. Intravenous calcium replacements were instituted for patients with symptomatic hypocalcemia or patients with a corrected calcium level < 1.9 mmol/L.

### Data collection

Ethics approval was obtained from the institutional internal review board (University of Western Ontario, REB# 105293). Consecutive HSRF patients who underwent parathyroidectomy with the SPARE technique between 2008 – July 2014 years were included. All operations were performed at Victoria Hospital, London Health Sciences Centre. Clinical and laboratory information was collected retrospectively. Recurrent disease was defined as patients who developed recurrent symptoms or increasing PTH levels refractory to medical management.

### Statistical analysis

Statistical analysis was performed with SPSS ver.20.0. Paired student t-test was used to compare change in value. A *p*-value of less than 0.05 was considered statistically significant.

## Results

During the study period, 30 consecutive patients with HSRF underwent parathyroidectomy with the SPARE technique. The mean surgical time was 92.4±21.4 min. There were 13 males and 17 females with a mean age of 53.1±12.5 years. The median follow up time was 17.1 months (range 1-78.9 months). Twenty-two (73.3%) patients were on haemodialysis at the time of surgery, 2 (6.7%) on peritoneal dialysis, and 6 (20.0%) were referred following renal transplantation (Table 1).

**Table 1:** Clinical summary of the 30 patients in this cohort

Clinical Information		
Number		30
Age		53.1±12.5
M:F		13:17
Hyperparathyroidism	Secondary	24
	Tertiary	6
Length of time on renal replacement therapy prior to surgery		Median 54.9 3.1 – 431.1 months
Renal support status at time of surgery	Haemodialysis	22
	Peritoneal	2
	Renal Transplant	6
Follow up		Median 17.1 months 1.0 – 78.9 months
Recurrence		3 cases

The cause of renal failure for this cohort is summarized in Table 2. The common indications for parathyroidectomy were elevated PTH (*n* = 30, 100%) and 5 patients with concurrent calciphylaxis. The mean pre-operative calcium and PTH levels were 2.5±0.5 mmol/L and 158.4±109.4 pmol/L respectively; and the mean post-operative levels were 2.1±0.3 mmol/L and 11.4±12.1 pmol/L respectively (Table 3).

**Table 2:** Causes of renal failure amongst the 30 patients

Causes of renal failure	Numbers
Alport syndrome	1
Bilateral nephrectomy for carcinoma	1
Congenital renal dysplasia	1
Diabetes Type I	3
Diabetes Type II	6
Glomerulosclerosis	1
Glomerulonephritis	3
Hypertension^a^	3
IgA nephropathy	2
Lithium^a^	2
Nephrotic syndrome	1
Obstructive nephropathy	1
Polycystic kidney disease	3
Reflux nephropathy	1
Rhabdomyolysis	1

^a^denotes patients who also have type II diabetes as contributing factors

**Table 3:** Biochemical profiles of the 30 patients

	Pre-operative (Mean ± SD)	Post-operative (Mean ± SD)	6 Months Post-operative (Mean ± SD)
Calcium (mmol/L) Normal range: 2.15 – 2.55	2.5±0.5	2.1±0.3^†^	2.2±0.3^†^
Phosphate (mmol/L) Normal range: 0.8 – 1.33	1.7±0.7	1.6±0.6	1.7±0.7
PTH (pmol/L) Normal range: 1.6-6.9	158.4±109.4	11.4±12.1^†^	18.4±31.4^†^
ALP (U/L) Normal range: 36 -104	300.0±333.2	231.3±182.8	116.8±152.8^†‡^

^†^ *p* < 0.05 when compared to pre-operative value

^‡^ *p* < 0.05 when compared to post-operative value

### Operative findings

Four parathyroid glands were found in all 30 parathyroidectomies. The mean total weight of the glands were 2.7±1.7 g and the average time of surgery was 92.4±21.4 min. Twenty patients underwent single gland remnant relocations with 6 upper glands and 14 lower glands relocated. In 9 patients, both lower glands were relocated, whilst 1 patient had both upper glands relocated. Perforations of the strap muscles were performed in 4 patients, of which 3 were for relocation of upper gland and 1 was for relocation of the lower gland. Three hemithyroidectomies were performed, of which 2 were performed for short vascular pedicle of the relocated parathyroid glands and 1 was due to abnormally appearing nodule (Table 4). None of these patients required thyroid hormone replacement when reviewed after 1 year postoperatively.

**Table 4:** Information on parathyroid gland relocation procedure

Operative Information		
Mean operative time		92.4±21.4 min
Parathyroid gland relocation	Right upper	4
	Right lower	9
	Left upper	2
	Left lower	5
	Both upper	1
	Both lower	9
Additional manoeuvres	Strap perforations	4 (3 for the upper glands)
	Sandwich between strap/SCM	2 (both for the upper glands)
Thyroid gland removal		2 for access 1 due to dominant nodule

*SCM* Sternocleidomastoid muscle

There were no RLN injuries or neck hematomas. One patient developed tetany and one neuromuscular hyper-reactivity due to hypocalcaemia. Thirty days mortality occurred in one patient due to acute myocardial infarction on post-operative day 4. This was not presumed related to serum calcium imbalance.

### Re-exploration

During the follow-up period, 3 recurrences were seen. The mean time to recurrence was 15.3±6.6 months. Of these, 2 underwent successful re-exploration, while one patient is currently being medically managed but considering re-operation. One re-exploration was performed under local anaesthetic with biochemical resolution of the PTH level (53 pmol/L to 10.7 pmol/L). The remnant hyperplastic parathyroid tissue was found at the site of initial placement under the skin incision. The total operative time was 29 min. The other re-exploration was conducted for a suspected recurrence within the thymus since only a small superficial remnant was seen overlying the strap muscles on ultrasound imaging. Re-exploration was performed under general anaesthetic with removal of the remnant hyperplastic parathyroid tissue, removal of thymic contents and exploration of the carotid sheath/lateral neck. No complications occurred in both cases.

## Discussion

This study described a modified surgical technique based on the conventional STP for patients with HSRF. The SPARE technique was developed in order to incorporate many of the advantages of the STP and TPAT, while mitigating the disadvantages of each. As this technique is a modification of the existing STP technique, it does not change the routine approach for the operation for surgeons who are familiar with STP.

Comparable biochemical outcomes were achieved with SPARE versus conventional STP, with postoperative PTH levels of 11.1±12.2 pmol/L [7]. Both STP and TPAT offer similar early results for patients with HSRF. However, patients with TPAT may have increase likelihood of recurrence as compared to STP [12]. Furthermore, eradication of hyperplastic parathyroid tissue within the site of autotransplantation may be extremely problematic [15]. Three of 30 patients recurred in our series, with two successful re-explorations and one managed medically. Of the 2 re-explorations, one of them was performed under local anaesthetic.

Proponents of TP posit reduced recurrence rates as compared to both STP and TPAT [19]. However, TP is clearly associated with a higher incidence of permanent hypoparathyroidism with rates as high as 20% and the management of calcium homeostasis may be more difficult in the postoperative setting [7, 16–18, 21–23]. TP may be a reasonable option for critically ill patients or when renal transplantation is no longer an option [24]. Furthermore there may be inherent benefits of retaining normal postoperative PTH levels even in patients who remain dialysis dependent. A recent publication by Fotheringham et al. showed that very low level of PTH post-operatively amongst patient with HSRF was associated with decreased survival [25].

Thymectomies were not routinely performed in our patients. Although the incidence of ectopic parathyroid glands within the thymus has been shown to be 5-20%, the benefit of routine thymectomies remains controversial [1, 7, 26, 27]. In our practice, we consider thymectomy if less than 4 parathyroid glands were found on initial exploration, pre-operative imaging suggesting hyperplastic parathyroid tissue within the mediastinum, or in selected recurrent cases of renal hyperparathyroidism. In this series, one patient failed at the mediastinum. In this patient, given that the surgical plane around the thymic tissue remained intact during the initial exploration, the access to this plane was straight forward during the re-exploration with no additional morbidities to the RLN. Nevertheless, for surgeons who offer thymectomy as part of the initial operation, incorporating the SPARE technique is still possible.

Recurrence following parathyroidectomy for HSRF is a well-recognized problem and should be anticipated to occur in a significant number of patients who continue dialysis. Each surgical technique has distinct advantages and disadvantages in regards to recurrence rates and complications associated with revision surgery. In balancing these variables, STP has become the most common operation for HSRF patients, and especially when patients are candidates for renal transplantation. The SPARE operation does not reduce recurrence rates compared to conventional STP nor does it alter the challenges related to mediastinal recurrence. The recurrence rate in our cohort was 10%, which is comparable to the other series of conventional STP or TPAT [7, 19, 28]. However the SPARE offers clear advantages over conventional STP in situations where re-exploration is required due to the retained remnant parathyroid gland, which is conventionally situated within the thyroid bed [4, 29]. With the SPARE technique, the remnant parathyroid gland is placed in an easily accessible location superficial to the strap muscles and under the skin closure. Re-exploration and removal of this remnant tissue may be safely performed and may be done without a general anaesthetic.

## Conclusion

We described the SPARE technique of parathyroidectomy for HSRF, as a modification of the standard STP operation and report our results in 30 consecutive patients. This surgical approach was shown to achieve similar results compared to the conventional approach while enabling easier and safer access to the remnant in cases of recurrent disease.

## Abbreviations

HSRF: Hyperparathyroidism secondary to renal failure
PTH: Parathyroid hormone
RLN: Recurrent laryngeal nerve
SPARE: Subtotal parathyroidectomy and remnant relocation
STP: Subtotal parathyroidectomy
TP: Total parathyroidectomy without autotransplantation
TPAT: Total parathyroidectomy with autotransplantation
